# Mapping the RNA methylome under drought: techniques, mechanisms, and agricultural implications

**DOI:** 10.3389/fpls.2026.1766123

**Published:** 2026-02-16

**Authors:** Xiaoru Fan, Yong Zhang

**Affiliations:** 1 School of Chemistry and Life Science, Anshan Normal University, Anshan, China; 2 Liaoning Key Laboratory of Development and Utilization for Natural Products Active Molecules, Anshan, China; 3 Wuxi Branch of Jiangsu Academy of Agricultural, Wuxi, China

**Keywords:** crop improvement, drought stress, epitranscriptomics, N6-methyladenosine, RNA methylation

## Abstract

Drought stress is one of the most devastating threats to global agriculture. Understanding plant adaptation to water scarcity is of paramount importance for food security. In the last several years, epigenetic regulation, especially RNA methylation, has been shown to play an important role in post-transcriptional gene regulation in plant stress response. Here, we summarize recent advances in studying the epitranscriptomic mechanisms underlying plant drought tolerance. We will introduce various types of RNA modifications, provide an overview of “writer”, “eraser” and “reader” proteins mediating m6A modification in plant system, and discuss different technologies for detecting m6A and several other modifications including m5C, m1A, m3C, m7G and m1A with focus on principles and technical consideration. Finally, we will discuss evidence from multiple species to suggest that water deficiency can alter the abundance of m6A modification on RNA molecules in a dynamic manner. The modified transcripts go through differential stability, translation efficiency and process proficiency levels to regulate various physiological processes including but not limited to stomatal movement, ROS signaling and hormonal action. Furthermore, we will also highlight the possible means through which modulation of m6A level could be utilized for generating drought tolerant crops through genetic or biotechnological approaches. This analysis establishes RNA methylation, particularly m6A, as a pivotal and reversible regulatory mechanism in plant drought stress responses and identifies key future research avenues for both fundamental understanding and crop improvement.

## 1 Introduction

Drought is one of the most threatening abiotic stresses facing crops globally, and drought stress greatly reduces crop quality and yield (Zhang et al., 2023). Due to increased evapotranspiration, water scarcity will become more severe with global warming, and the annual crop yield reductions due to drought factors around the world are enormous and have exceeded the yield reductions due to other environmental factors combined (Lesk et al., 2016). Drought tolerance has been identified as a major concern for crop improvement due to the increasing problems of decreasing water resources and arable land constraints (Shan et al., 2018; Fan et al., 2024). Drought tolerant utilization of crops and development of marginal land for cultivation is gradually becoming imperative (Shan et al., 2018). Drought stress leads to a series of physiological changes in plants such as restricted leaf stomatal conductance (Lawlor and Tezara, 2009), reduced CO_2_ uptake, limited carbon assimilation processes, unsteady light energy utilization and altered chloroplast photochemistry, which leads to a decrease in photosynthetic rate (Ramachandra Reddy et al., 2004; Mukarram et al., 2021).

Since plants are a diverse group of organisms that cannot move from their location, they need to develop strategies to cope with changing environments without compromising their ability to survive and reproduce in the future (Alonso et al., 2019). Epigenetic regulation is closely linked to plant response to drought stress. The role of epigenetic factors, including DNA methylation, small RNAs regulation, post-translational histone modifications and RNA modification has become significant in modulating plant responses to the environment (Lamke and Baurle, 2017). Among epigenetic modifications, methylation modifications are present on DNA, RNA and histones (Shinde et al., 2023). Therefore, this review summarizes the latest research advances in RNA methylation under drought stress, focusing on its modification types, detection techniques, and regulatory mechanisms. Unlike previous reviews that primarily focused on the mechanisms of m6A in stress within single species, this review integrates dynamic changes in RNA methylation profiles across diverse crop and forest tree species under drought stress. It systematically reviews detection technologies applicable to plant samples and highlights the translational potential of epigenomics in crop improvement. By synthesizing cross-species evidence and integrating mechanistic insights with agricultural applications, this review aims to provide a timely and targeted academic resource to advance both theoretical understanding and practical strategies for enhancing crop drought tolerance.

## 2 RNA methylation types and features

RNA modification, a type of nucleic acid modification at the post-transcriptional level, has been a hot research topic in the field of epigenetics in recent years (Chmielowska-Bak et al., 2019). More than 150 RNA modifications have been identified, and they are widely distributed on different classes of RNAs such as messenger RNA (mRNA), transporter RNA (tRNA), ribosomal RNAs (rRNA) and other non-coding RNA (ncRNA) (Nachtergaele and He, 2017). The most abundant internal RNA modification is methylation of RNA. Methylation of RNA means that methylation is formed at specific sites on RNA bases, such as nitrogen and carbon atoms. The types of RNA methylation modifications widely found in eukaryotes include N6-methyladenine (m6A), 5-methylcytosine (m5C), 3-methylcytosine (m3C), N7-methylguanine (m7G), as well as N1-methyladenosine (m1A), N6,2-O-dimethyladenosine (m6Am) in mRNA. Meanwhile, Eukaryotic tRNAs contain base and ribose methylations, and human rRNA contains 2-O-methyl and base methylations (Roundtree et al., 2017).

N6 -Methyladenine (m6A) refers to the methylation of adenosine nucleotides at the N-6 position and is the most common and most abundant form of methylation modification in eukaryotic mRNAs (Kierzek and Kierzek, 2003; Roundtree and He, 2016). m6A modification was first identified in Novikoff hepatocellular carcinoma cells in 1974 (Desrosiers et al., 1974). Subsequently, m6A modifications have been found in mouse (Mus musculus) (Schibler et al., 1977), Drosophila (Drosophila melanogaster) (Levis and Penman, 1978), wheat (Triticum aestivum) (Kennedy and Lane, 1979), oat (Avena sativa) (Haugland and Cline, 1980), corn (Zea mays) (Nichols and Welder, 1981), yeast (Saccharomyces cerevisiae) (Bodi et al., 2012), bacteria (Deng et al., 2015) and viruses (Courtney et al., 2017). m6A modifications account for more than 80% of all RNA methylation modifications (Kierzek and Kierzek, 2003). In yeast, the relative amount of m6A [(m6A)/A] is 0.7% to 0.9% (Bodi et al., 2010), whereas in animals and Arabidopsis thaliana, the relative amount of m6A ranges from 0.1% to 0.4% and 0.4% to 1.5%, respectively (Zhong et al., 2008; Shi et al., 2017). Sequences in the vicinity of the m6A modification site have preference and are mainly present on RRACH (R: purine; A: m6A; H: non-guanine) in mammal (Dominissini et al., 2012). In plants, such as Arabidopsis thaliana and maize, in addition to the RRACH sequence, m6A is also distributed in plant-specific UGUA sequences (Luo et al., 2014; Wei et al., 2018; Miao et al., 2020). m6A modifications are distributed in various regions of mRNA, including near the 5’UTR (untranslated region), CDS (coding sequence), 3’UTR, termination codon, and the transcription start site (TSS), and are highly enriched in the vicinity of the termination codon and within the 3’ UTR (Dominissini et al., 2012; Meyer et al., 2012; Zhou et al., 2021).

**Figure 1:**
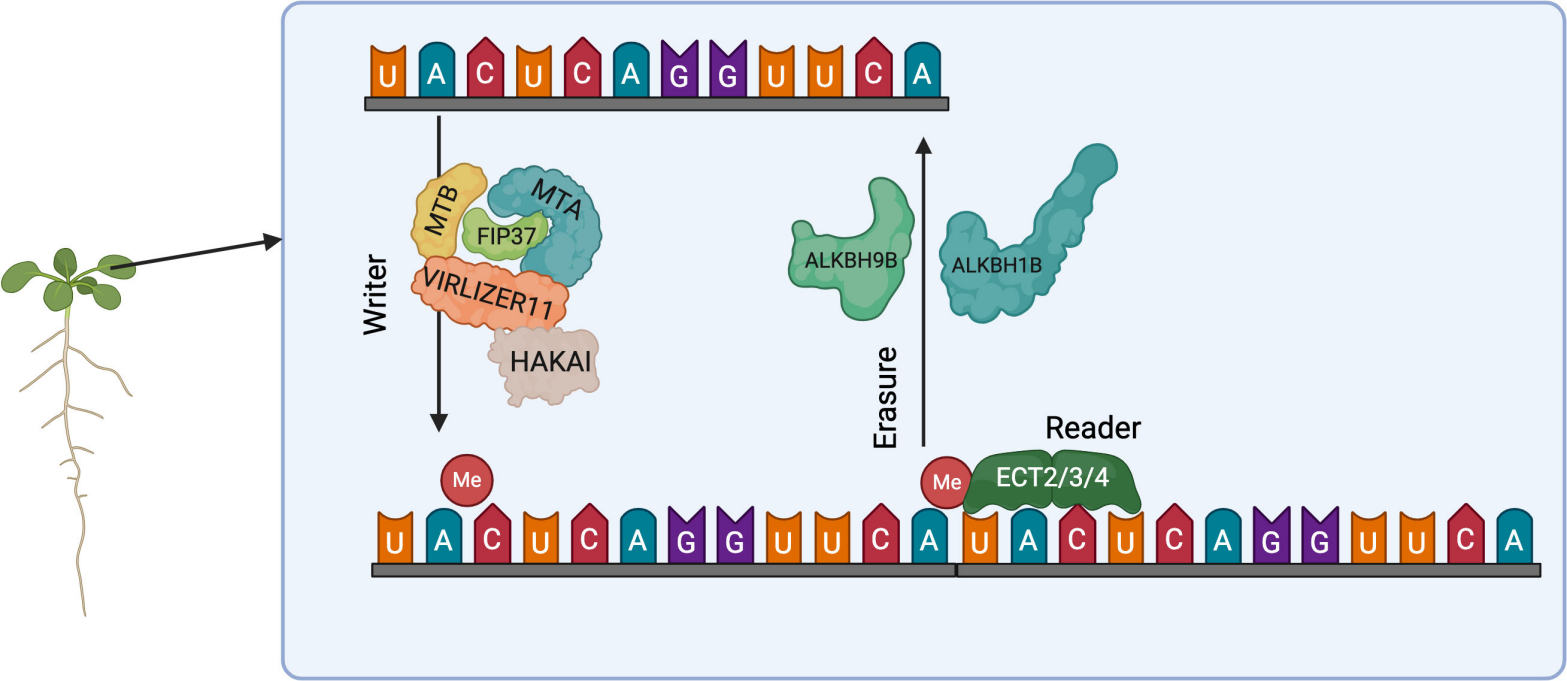
RNA N6-methyladenine in arabidopsis.

Eukaryotic mRNAs are methylated by m6A methyltransferases, which recognizes and methylates target mRNAs as a complex (Liu et al., 2014). Writing of m6A is accomplished by a methyltransferase complex. In animals, the complex consists of methyltransferase like 3 (METTL3), methyltransferase like 14 (METTL14), Wilms’ tumor 1-associating protein (WTAP), KIAA1429 (also known as VIRMA: vir-like m6A methyltransferase-associated protein) (Ping et al., 2014; Schwartz et al., 2014). In Arabidopsis thaliana, m6A methyltransferase components include mRNA adenosine methylase (MTA, METTL3 human homolog), MTB (METTL14 human homolog), FKBP12 interacting protein 37 (FIP37, WTAP human homolog), VIRILIZER (KIAA1429 human homolog), and the E3 ubiquitin ligase HAKAI (Zhong et al., 2008; Shen et al., 2016; Ruzicka et al., 2017). The m6A methyltransferase complex catalyzes the addition of methyl groups to adenosine residues at the N6 position using S-adenosylmethionine (SAM) as the methyl donor. This writer complex specifically recognizes and binds to conserved RNA motifs, such as RRACH sequences (where R = purine, A = adenine, C = cytosine, H = non-guanine), to achieve site-specific methylation, thereby establishing the m6A landscape critical for post-transcriptional regulation of gene expression during development and stress responses (Shen et al., 2016). Erasure of m6A is accomplished by demethylases. The demethylases OBESITY-ASSOCIATED PROTEIN (FTO) and ALKBH5 (alkB, alkylation repair homolog 5) have been identified in animals (Jia et al., 2011; Jia et al., 2013; Zheng et al., 2013). In Arabidopsis, the demethylases Alpha-ketoglutarate-dependent dioxygenase homolog 9B (ALKBH9B) and ALKBH10B, homologous to ALKBH5, have been shown to be able to remove m6A methylation modifications (Duan et al., 2017; Martinez-Perez et al., 2017). RNA methylation recognition is mediated by reader proteins, predominantly YTH-domain proteins such as like EVOLUTIONARILY CONSERVED C-TERMINAL REGION2/3/4 (ECT2/3/4). These readers specifically bind to m6A-modified RNAs through their conserved YT521-B homology (YTH) domains, orchestrating downstream regulatory outcomes (Liao et al., 2018). For instance, they modulate RNA stability by recruiting deadenylase complexes like CCR4-NOT, which catalyze poly (A) tail removal to promote RNA degradation (Du et al., 2016). At the same time, readers can also increase the translation efficiency by either promoting ribosome loading on the target mRNAs or help in the formation of polysomes, thereby increasing protein synthesis. Additionally, they regulate subcellular localization of methylated RNAs, either by mediating nuclear-cytoplasmic shuttling or participating in phase separation events that compartmentalize RNAs into membraneless organelles (e.g., stress granules). This multifaceted control ensures precise spatiotemporal regulation of RNA function in response to developmental cues or environmental stresses (Arribas-Hernandez et al., 2018; Wei et al., 2018; Hou et al., 2021). The coordinated actions of these writer, eraser, and reader proteins in shaping the Arabidopsis m6A methylome and regulating RNA fate are schematically summarized in **Figure 1**.

## 3 RNA methylation detection methods

### 3.1 m6A detection techniques

Since the methylated base A has not changed its base pairing ability, it cannot be detected by direct sequencing. Unlike DNA methylation, the 5mC in DNA will undergo a base change after bisulphide treatment and can be detected by sequencing, m5A has not found to have a unique chemical reaction (Fan et al., 2020). Early detection relied on techniques like thin-layer chromatography (Keith, 1995) and dot blotting (Nagarajan et al., 2019; Du et al., 2022), or by tracking radiolabeled methyl donors (Munns and Liszewski, 1977; Canaani et al., 1979).

With the development of second-generation sequencing technology, two m6A high-throughput sequencing technologies were developed by two research teams in 2012 independently, called m6A-seq (Dominissini et al., 2012) and MeRIP-seq (m6A-specific methylated RNA immunoprecipitation with next-generation sequencing) (Meyer et al., 2012). Both methods involve immunoprecipitation (IP) and m6A-specific antibodies, followed by sequencing of about 100 nucleotide RNA fragments. They provide transcriptome-wide profiles of m6A-enriched regions, with MeRIP-seq typically focusing on mRNA and m6A-seq on total RNA (Meyer and Jaffrey, 2017). Researchers have characterized m6A modifications in Arabidopsis (Luo et al., 2014; Wang et al., 2015; Shen et al., 2016), rice (Li et al., 2014; Zhang et al., 2019), tomato (Zhou et al., 2019), strawberry (Zhou et al., 2021), and maize (Luo et al., 2020) with MeRIP-seq or m6A-seq. However, their resolution is limited to 100–200 nucleotide fragments, preventing precise site identification.

To achieve single-nucleotide resolution, methods like methylation-iCLIP (miCLIP) were developed. Its principle relies on cross-linking RNA-m6A antibody binding sites, which induces mutations at these specific sites when the antibody-bound RNA is reverse transcribed. Sequencing the unique mutational signature allows precise localization of m6A (Linder et al., 2015; George et al., 2017a). While offering single-nucleotide resolution, miCLIP infers m6A sites indirectly from mutations in neighboring pyrimidines. Consequently, it cannot accurately localize m6A within clusters of adjacent adenines, analyzing m6A distribution in such regions (Tang et al., 2023).

m6A-CLIP (Crosslinking and Immunoprecipitation) sequencing combines CLIP technology with an m6A antibody. RNA is crosslinked to the antibody via UV, followed by immunoprecipitation and sequencing. This method can study the interaction between m6A and RNA-binding proteins. However, it has limitations including low resolution (~100 nt), reliance on antibodies, and potential for false-positive results (Ke et al., 2015).

m6A-REF-seq (RNA Editing-based m6A Detection) is the deamination of unmethylated adenosine (A) to inosine (I) by nitrite treatment, which reads as G in sequencing, while the m6A remains unchanged (still as A). The A-to-G mutation is utilized to identify the m6A site (Schaefer et al., 2009). The advantage is that single bases can be resolved and the level of m6A modification can be quantified. Limitations is dependent on chemical processing, which may affect RNA integrity (Chen et al., 2022). Liu et al. provided GLORI-seq (Glyoxal and nitrite-mediated deamination of unmethylated adenosine) as an improved version of m6A-REF-seq, can perform m6A site detection with higher accuracy (Liu et al., 2023).

DART-seq (Deamination Adjacent to RNA Modification Targets) utilizes the APOBEC1-YTH fusion protein to deaminate cytosine (C) to uracil (U) in the vicinity of m6A, which reads as T in sequencing, thereby inferring the m6A site. It is cost-effective, antibody-free, and suitable for low RNA input, but results depend on transfection efficiency, limiting its use to certain cell types (Meyer, 2019; Saikia et al., 2019).

m6A-SAC-seq (Selective Allyl Chemical Labeling) labels m6A as N6-allyl-m6A (am6A) using the methyltransferase MjDim1. Subsequent cyclization of am6A with iodine treatment introduces a mutation (A→T/C) during reverse transcription to detect m6A. m6A-SAC-seq is single-base resolution, which can be quantitatively determined with only 30 ng of RNA. However, because it relies on enzymatic reactions, it may have sequence preferences (Hu et al., 2022).

Nanopore (m6Anet) is a direct RNA sequencing method. Because modified bases cause changes in current, this method identifies m6A by differences in current signals. nanopore detects single molecules of m6A without the need for antibodies or chemical treatments but relies on computational models and requires high sequencing depth (Hendra et al., 2022).

MAZTER-seq (MazF-mediated m6A Sequencing) takes advantage of that MazF endonuclease can cleave unmethylated ACA sites, leaving the m6A-modified m6ACA to be sequenced. Therefore, this method does not require antibodies. However, this method can only detect about 20% of the m6A sites (ACA motifs) (Garcia-Campos et al., 2019). For this reason, MAZTER-seq is only suitable for studying the distribution of m6A in specific RNA regions like 3’UTR. A systematic comparison of the principles, resolutions, advantages, and limitations of these key m6A detection methods is provided in **Table 1**.

**Table 1:** Comparison of m6A detection methods.

Method	Principle	Resolution	Advantages	Limitations	Reference
MeRIP-seq (m6A-Seq)	Antibody-based enrichment of m6A-modified RNA fragments followed by sequencing.	100–200 nt	Transcriptome-wide profiling. Established protocol	Low resolution. Antibody bias. Cannot quantify m6A stoichiometry.	(Dominissini et al., 2012)
miCLIP	UV crosslinking of anti-m6A antibody to RNA, inducing mutations at m6A sites.	Single-nucleotide	Single-base resolution. Identifies exact m6A sites.	Technically challenging. Low throughput.	(Linder et al., 2015)
m6A-CLIP	Crosslinking and immunoprecipitation of m6A-RNA-protein complexes.	~100 nt	Detects m6A sites bound by reader proteins.	Lower resolution than miCLIP. Antibody bias false-positive results.	(Ke et al., 2015)
m6A-REF-seq	Enzymatic deamination (m6A → T) followed by sequencing to detect mutations.	Single-nucleotide	High resolution. Quantitative.	Requires specific enzyme treatment.	(Chen et al., 2022)
DART-seq	APOBEC1-mediated C-to-U editing at m6A-adjacent sites.	Single-nucleotide	No antibody needed. Works with low input RNA.	Limited to DRACH motifs	(Meyer, 2019; Saikia et al., 2019)
m6A-SAC-seq	Chemical labeling of m6A (allyl tagging) followed by mutation detection.	Single-nucleotide	Quantitative. Works with low RNA input.	Requires chemicalmodification steps	(Hu et al., 2022)
Nanopore (m6Anet)	Direct RNA sequencing detecting current deviations caused by m6A.	Single-nucleotide	Detects m6A at single-molecule level. No antibody/chemical steps.	Computational complexity. Requires training data.	(Hendra et al., 2022)
MAZTER-seq	MazF ribonuclease cleavage at unmethylated ACA sites	Single-molecule	Quantitative. No antibody needed.	Limited to ACA.	(Garcia-Campos et al., 2019)

### 3.2 Detection of other RNA modifications

The development of m6A detection methods has advanced epitranscriptomic research and provided a framework for studying other RNA modifications. Based on principles such as immunoprecipitation and nucleotide-resolution mapping established for m6A, similar approaches have been adapted for other modifications.

For m5C, bisulfite sequencing achieves single-nucleotide resolution by deaminating unmodified cytosine, though it causes significant RNA degradation (Schaefer et al., 2009). Alternative methods like DRAM-seq improve specificity by using a fusion of a deaminase with an m5C reader protein (Weigel, 2024). m5C-RIP/miCLIP-seq offers transcriptome-wide profiling through antibody-based enrichment. miCLIP providing near-base resolution via crosslinking-induced mutation signatures (George et al., 2017b; Gu and Liang, 2019).

The identification of m3C is fundamentally different from bisulfite. By using the hydrazine-aniline cleavage in the HAC-seq procedure to specifically fragment RNA at the m3C locus, the modified cytosine can be directly and resolutionly mapped in a chemically specific manner (Cui et al., 2021).

Mapping m7G in the transcriptome with m7G-MaP-seq. This method makes use of mild reduction to transform m7G to an abasic web site, which will induce signature mutations throughout reverse transcription, for transcriptome-wide, single-nucleotide decision profiling (Enroth et al., 2019).

The red-m1A-seq/m1A-ID-seq assay uses an entirely chemical-reduction-based detection and a controlled alkaline rearrangement protocol to create a reverse transcription signature that is verifiable by the sequencing technology (Li et al., 2017). Although nanopore direct RNA sequencing is a single, antibody-free method for simultaneous detection of m1A and other modifications by analyzing native RNA molecule current signatures, it does require computational expertise (Chen et al., 2024).

Distinguishing the cap-proximal modification m6Am from internal m6A can be discriminated by m6Am-seq, where the m6Am is selectively demethylated to an m6A, immunoprecipitated as m6A and subjected to sequencing (Sun et al., 2021). The techniques for identifying these different RNA modifications are summarized in **Table 2**.

**Table 2:** Methods for detecting other RNA modifications.

Method	Modification type	Principle	Resolution	Advantages	Limitations	Reference
DRAM-seq	m5C	Fusion of a deaminase with an m5C reader protein for targeted recognition and conversion.	Single-nucleotide	High specificity. Low RNA input. Less RNA degradation.	Requires specific reader protein.	(Weigel, 2024)
Bisulfite-seq (RNA)	m5C	Bisulfite treatment converts unmodified C to U, while m5C remains unchanged.	Single-nucleotide	Gold standard. High accuracy for base. Resolution mapping.	Causes severe RNA degradation. Complex data analysis.	(Schaefer et al., 2009)
m5C-RIP-seq/miCLIP	m5C	Immunoprecipitation using antibodies specific to m5C.	100–200 nt (RIP)/~Single-nucleotide (miCLIP)	Suitable for transcriptome-wide profiling. miCLIP offers near-base resolution.	Antibody-dependent specificity and bias. Lower resolution.	(George et al., 2017b; Gu and Liang, 2019)
HAC-seq	m3C	Hydrazine specifically reacts with m3C under high-salt conditions, followed by aniline-induced cleavage at the modification site.	Single-nucleotide	High chemical specificity for m3C. Provides direct mapping.	Requires specific chemical reaction.	(Cui et al., 2021)
m7G-MaP-seq	m7G	Mild reduction converts m7G to an abasic site, inducing characteristic mutations during reverse transcription.	Single-nucleotide	Enables transcriptome-wide Mapping at base resolution.	Background noise.	(Enroth et al., 2019)
Nanopore Direct RNA Sequencing	m1A	Directly passes native RNA through a nanopore; modifications alter the electrical current signature, which is decoded by base-calling algorithms.	Single-nucleotide (Single-molecule)	No chemical conversion or RT.	Specialized equipment and trained models. Lower throughput/accuracy.	(Chen et al., 2024)
red-m1A-seq/m1A-ID-seq	m1A	Chemical reduction stabilizes m1A, enhancing reverse transcription signature, combined with alkaline rearrangement control.	Single-nucleotide	High sensitivity and accuracy.	Involves multiple chemical treatment steps.	(Li et al., 2017)
m6Am-seq	m6Am	Selective *in vitro* demethylation to convert m6Am to m6A, followed by MeRIP)to distinguish it from internal m6A.	Single-nucleotide	♦ Specifically discriminates m6Am from internal m6A sites.	• Specific enzymatic activity and antibody efficiency.	(Sun et al., 2021)

## 4 The regulatory role of m6A in plant drought stress responses

RNA methylation, especially m6A, as post-transcriptional regulators play a central role in plant responses to drought stress. It dynamically controls mRNA stability, translation and processing to fine-tune the expression of stress-responsive genes.

The functional outcomes of m6A modification are executed by a coordinated set of proteins: writers (methyltransferases), erasers (demethylases), and readers (binding proteins). Their activities collectively shape the dynamic m6A landscape under stress. Transcriptome-wide analyses across diverse species have mapped the global m6A landscape under drought stress, revealing species and context-specific patterns that reflect the actions of these regulatory proteins. In cotton, m6A is generally enriched in 3’UTRs. Drought-treated cotton plants have increased global m6A content, and drought-tolerant cotton varieties accumulate more m6A in 5’UTRs than drought-sensitive ones (Sun et al., 2021). In wheat, drought stress causes 4221 differentially expressed m6A peaks that are mainly enriched in 3’UTRs and positively correlated with gene expression level (Linder et al., 2015). In sugarcane, whole-transcriptome m6A sequencing analysis revealed that drought stress significantly increases m6A modifications on transcripts associated with stress responses. This modification finely regulates gene expression by enhancing mRNA stability, such as in pathways involved in abscisic acid (ABA) biosynthesis (Wei et al., 2023). However, drought stress can globally decrease the levels of m6A modification in Arabidopsis thaliana and sea buckthorn by up-regulating m6A demethylase gene expression, which is consistent with this contradiction due to different species-specific adaptation strategies or at different stress time points (Huong et al., 2020; Miao et al., 2020). In poplar trees, drought can increase the levels of m6A methylation near stop codons and utilizes distal poly (A) sites for mRNA degradation of secondary cell wall synthesis-related genes (Han R. et al., 2023). A combined epitranscriptomic and proteomic study further revealed that these drought-induced m6A changes in stem-differentiating xylem are closely linked to the suppression of genes involved in wood formation, highlighting a direct role of m6A in reprogramming developmental processes under stress (Gao et al., 2022). Beyond poplar, research in rice indicates that drought influences the m6A modification landscape of polysome-bound mRNAs, thereby modulating translational efficiency and presenting a crucial layer of regulation in drought response (Dawane et al., 2024). Furthermore, genome-wide analysis in switchgrass revealed that drought stress significantly alters the expression profiles of m6A-regulated genes, with particularly pronounced downregulation of reader genes. This suggests that this protein category may play a specific regulatory role in stress adaptation (Liu et al., 2024).

The writers, such as MTA in Arabidopsis, are critical for drought stress, as its loss leads to hypersensitivity. MTA-dependent m6A methylation enhances translation of drought inducible gene RD29A and COR47 (Wu et al., 2024). Writer function is conserved across species to improve drought resistance, as evinced by enhanced water melon drought resistance mediated by ClMTB, NtFIP37B of tobacco, PbrMTA1 in pear and PtrMTA of poplar (He et al., 2021; Li et al., 2023; Luo et al., 2023; Kumari et al., 2024). Specifically, in pear trees, silencing the m6A methyltransferase gene PbrMTA1 reduces plant drought tolerance, further confirming the crucial role of the “writer” function in drought resistance of woody fruit trees (Han C. et al., 2023). MdMTA improves apple’s resistance stabilizing the transcripts involved in lignin deposition and oxidative stress (Zhang et al., 2021).

Readers recognize the m6A mark to execute downstream processes. The reader protein ECT8 in Arabidopsis is an intracellular sensor of ABA, and forms phase-separated condensates that feed back to ABA signaling and drought response regulation (Yu et al., 2021). Biomolecular condensates formed through phase separation have emerged as a novel mechanism by which m6A effectors regulate RNA metabolism and mediate plant stress adaptation (Shen, 2025). In foxtail millet, the reader SiYTH1 imparts drought tolerance by stabilizing mRNAs that regulate stomatal closure and ROS scavenging; loss-of-function mutants are drought-sensitive while overexpressors are tolerant (Pan et al., 2024). The wheat reader TaETC9 is also required for full drought tolerance (Linder et al., 2015). Transcriptomic studies in pine and camellia also show that reader genes are responsive to drought, pointing towards their functional importance being conserved as well (Lu et al., 2020; Yao et al., 2024).

Erasers provide reversible control. The Arabidopsis demethylase ALKBH10B is a positive regulator of drought tolerance and seed germination during dehydration stress (Hou et al., 2022). Homologs of this enzyme, including GhALKBH10B in cotton, regulate drought response by decreasing m6A levels on ABA and Ca²^+^ signaling gene transcripts to accelerate their decay (Sun et al., 2021). In rice, analysis of the expression profiles of m6A-regulating genes revealed that different types of “eraser” and “writer” genes exhibit complex temporal expression changes under stresses such as drought (Hasan et al., 2024). Other erasers such as ALKBH6 and ALKBH9C in Arabidopsis can also affect drought resistance and seed germination, even though they may display complex and developmentally regulated effects (Mao et al., 2021). Drought strongly induces the expression of genes for demethylases like CcALKBH10B in pigeon pea and HrALKBH10B/C/D in sea buckthorn, emphasizing their wider involvement in responses to stress (Wei et al., 2023; Su et al., 2024).

The importance of the m6A pathway is further highlighted by recent success of biotechnological applications. Transgenic expression of the human RNA demethylase FTO in rice and potato plants increases root growth and photosynthesis, and enhances drought tolerance, underlining the biotechnological potential of utilizing epitranscriptomic information for crop improvement (Gao et al., 2022). In summary, these studies unequivocally demonstrate that m6A plays a central role in coordinating the intricate network of transcriptional and translational reprogramming for plants to cope with water-deficit stress. Compilation of evidence demonstrating the role of m6A writers, erasers and readers in drought tolerance from various plant species is provided in **Table 3**.

**Table 3:** Role of m6A in plant drought stress response.

Species	Gene/study type	Function	m6A detection method	Reference
Arabidopsis thaliana	ALKBH6	Acts as an m6A demethylase; mutant shows reduced survival under drought stress.	The EpiQuik™ m6A RNA Methylation Quantification Kit	(Huong et al., 2020)
Arabidopsis thaliana	ALKBH10B	Demethylase mutant is drought-sensitive, while overexpression enhances tolerance	/	(Han R. et al., 2023)
Arabidopsis thaliana	ECT8	An m6A reader protein that specifically binds to m6A-modified mRNA of ABA receptors, forming phase-separated condensates to feedback-regulate ABA signaling perception and drought stress response.	Dot blot analysis, LC–MS/MS/m6A-IP, RIP-seq, LC-MS/MS	(Wu et al., 2024)
Arabidopsis thaliana	Systemic Study	Drought upregulates demethylases, reducing global m6A levels. Salt stress induces m6A writers/erasers/readers, while drought mainly suppresses readers.	/	(Miao et al., 2020)
Cajanus (Pigeon pea)	CcALKBH10B	Demethylase gene shows strongest induction under drought stress.	/	(Kumari et al., 2024)
Cereals (Setaria italica)	SiYTH1	m6A reader protein; enhances drought tolerance by regulating stomatal closure and ROS scavenging. Mutants are drought-sensitive, while overexpression enhances tolerance.	/	(Luo et al., 2023)
Citrullus (Watermelon)	ClMTB	m6A methyltransferase; overexpression improves drought tolerance by enhancing ROS scavenging and photosynthesis.	/	(He et al., 2021)
Gossypium (Cotton)	GhALKBH10B	An m6A demethylase (eraser). Reduces m6A levels, promotes mRNA degradation of ABA/Ca²^+^ signaling genes, regulating drought tolerance in a Ca²^+^ and ABA-dependent manner. Mutation enhances drought tolerance at seedling stage.	MeRIP-seq	(Li et al., 2023)
Hippophae rhamnoides (Sea buckthorn)	HrALKBH10B/C/D	Drought increases demethylase expression, potentially reducing m6A levels.	m6A-seq	(Zhang et al., 2021)
Malus (Apple)	MdMTA	An m6A methyltransferase (writer). Enhances drought tolerance by mediating m6A modifications.	m6A-IP-qPCR/m6A-immunoprecipitation (IP)-qPCR	(Hou et al., 2022)
Malus (Apple)	(Transcriptome-wide)	Drought induces hypermethylated peaks; m6A regulates drought-responsive genes (HSP60, JAZ3, etc.).	MeRIP-seq	(Mao et al., 2021)
Nicotiana (Tobacco)	NtFIP37B	m6A writer gene; enhances drought resistance.	/	(Su et al., 2024)
Oryza sativa (Rice)/Solanum tuberosum (Potato)	FTO (Transgenic)	Ectopic expression of the demethylase improves drought tolerance by stimulating root development and photosynthetic efficiency.	m6A ELISA	(Yu et al., 2021)
Oryza sativa (Rice)	Systemic Study	Drought stress elevated m6A modifications.	m6A RNA Methylation Quantification Kit	(Pan et al., 2024)
	Systemic Study	Different types of writer and eraser genes exhibit complex temporal expression changes under drought stress, suggesting precise temporal regulation of the m6A pathway.	RNA-seq/RT-qPCR	(Dawane et al., 2024)
	Genome-wide Identification & Expression Profiling	Identified m6A RNA methylation genes in rice. Expression profiling reveals their differential regulation under various developmental stages and environmental stimuli, including abiotic stresses.	RT-qPCR	(Hasan et al., 2024)
Pinus (Pine)	PmALKBH4/6, PmYTHDF1/3	Stress expression analysis suggests m6A-regulated genes may function in drought response.	/	(Yao et al., 2024)
Populus trichocarpa (Poplar)	PtrMTA	An m6A methyltransferase (writer). Overexpression improves drought tolerance by affecting trichome and root development; part of the m6A methyltransferase complex.	Dot blot/Adelaide RNA Kit	(Lu et al., 2020)
	Transcriptome-wide	Drought increases m6A levels and usage of distal poly(A) sites, suppressing wood formation genes.	Nanopore Direct RNA-seq	(Gao et al., 2022)
Pyrus (Pear)	PbrMTA1	Silencing this m6A writer reduces drought resistance.	/	(Han C. et al., 2023)
Saccharum (Sugarcane)	Transcriptome-wide	Drought increases m6A modification of stress-responsive transcripts, enhancing mRNA stability.	MeRIP-seq	(Wei et al., 2023)
Triticum aestivum (Wheat)	TaETC9	An m6A reader. Knockout increases drought sensitivity. Drought stress induces 4,221 differentially expressed m6A peaks, enriched in 3′ UTRs.	MeRIP-seq	(Pan et al., 2024)
*Panicum virgatum* (Switchgrass)	16 writers, 17 erasers, 24 readers	Drought stress predominantly leads to the decreased expression of reader genes	RT-qPCR	(Liu et al., 2024)

## 5 Future perspectives

Progress has been made, but the epitranscriptomic regulation of plant responses to drought remains largely unexplored. Especially the field of non-m6A RNA methylation in drought response remains highly underexplored, presenting a critical knowledge gap and an exciting frontier for future research. Furthermore, more studies should focus on attaining spatiotemporal resolution of RNA modification dynamics in tissues, cell types, and compartments during stress progression, using emerging single-cell and spatial transcriptomics technologies. Understanding how the epitranscriptome crosstalks with other epigenetic processes, such as DNA methylation, histone modifications, and noncoding RNAs to establish the stress memory and coordinate the adaptive response represents a major challenge and opportunity.

Using genetic engineering, genome editing, and synthetic biology to precisely control the activity of key writer, eraser or reader proteins can offer an approach to engineer crop plants that are resilient to stress. However, translating this potential into reliable crop improvement requires careful navigation of several challenges. As observed in certain mutant studies, manipulation of m6A levels may lead to pleiotropic developmental defects (Shen et al., 2016), underscoring the necessity for precise and context-specific control as m6A inherently regulates a broad spectrum of growth and developmental processes alongside stress responses (Jia et al., 2013). Therefore, future research should explore stress-specific, spatially controllable regulatory strategies, such as employing stress-inducible promoters or tissue-specific regulatory systems. Furthermore, epigenomic engineering requires extensive testing across diverse genetic backgrounds and agronomic environments to balance stress tolerance with yield. Comprehensive field trials that evaluate changes in key yield components under realistic drought stress scenarios are indispensable for translating laboratory findings into tangible crop improvement.

Continued innovation in RNA detection methods, particularly to enhance their performance and standardization for plant samples, will be critical for uncovering new regulatory insights and translating mechanistic knowledge into crop improvement.
